# Amplitude of low frequency fluctuations (ALFF) of spontaneous and induced rumination in major depression: An fNIRS study

**DOI:** 10.1038/s41598-020-78317-y

**Published:** 2020-12-09

**Authors:** David Rosenbaum, Isabell Int-Veen, Agnes Kroczek, Paula Hilsendegen, Kerstin Velten-Schurian, Isabel Bihlmaier, Andreas J. Fallgatter, Ann-Christine Ehlis

**Affiliations:** 1 Department of Psychiatry and Psychotherapy, University Hospital of Tuebingen, Calwerstraße 14, 72076 Tübingen, Germany; 2 LEAD Graduate School and Research Network, University of Tübingen, Tübingen, Germany; 3 German Center for Neurodegenerative Disorders, Tübingen, Germany

**Keywords:** Human behaviour, Cognitive control, Depression

## Abstract

In the current study, we investigated the amplitude of low frequency fluctuations (ALFF) at rest and during a rumination induction. Specifically, we explored the differences of cortical blood oxygenation using fNIRS in subjects with Major Depressive Disorder (MDD) and healthy controls (HC). Rumination was assessed as state and trait measure, as well as with a qualitative semi-structured interview. Qualitative and quantitative measures of rumination indicated that the MDD group showed elevated rumination regarding state and trait measures. Furthermore, rumination differed qualitatively between the groups. The MDD group showed higher levels of general rumination and increased rumination during the rumination induction. However, the MDD group did not show a carry-over effect of elevated rumination after the induction paradigm to the following resting-state measurement. On a neuronal level, we observed a general hypoactivity in the MDD group compared to the HC group. Moreover, both groups showed increased ALFF during the rumination induction compared to the rest phase, especially in temporo-parietal areas. However, no interaction effect of MDD status and rumination induction was found. The current findings are discussed with respect to the literature of paradigms used in the investigation of rumination and suggestions on general improvements in rumination research are given.

## Introduction

Depressive rumination is defined as a perseverative, rather pessimistic, self-related cognitive process, focused on the past with little goal orientation based on negative affect^1,2^. Rumination is a form of repetitive negative though, i.e. perseverative cognition, such as worry and generally a rather natural cognitive process that is known to all humans. However, the level of rumination does differ significantly between healthy participants and patients with mental disorders and has a high clinical relevance^3–5^. Subjects with higher ruminative tendencies are more likely to develop depression, with longer episodes of disease, stronger symptom severity and higher relapse risk^1,2,6–10^. Although rumination has been investigated intensively due to its prominent role in the development and maintenance of major depressive disorder (MDD), the evidence regarding the neuronal correlates of depressive rumination is far from conclusive^11–14^. Investigations on the matter of depressive rumination have been done using experimental designs^15–19^ as well as resting state measurements^13,20,21^ with many dependent variables (e.g. activity and functional connectivity). Some part of the divergent evidence on the neuronal substrates of depressive rumination might be due to differences in design and induction. For example, in some cases spontaneous occurrence of rumination is investigated using resting-state measurements^13^, whereas in other research paradigms an indirect induction^22,23^ or direct induction methods are used^17,18,24^. We define direct induction methods as experimental designs that instruct the participants to think in a certain way, for example by reading words in a self-referential task^25–31^, or imagine a certain situation during which they ruminated^20^. In contrast, indirect induction methods induce rumination by creating a situation during which rumination might be triggered, e.g. through social stress or a specific emotional situation, and measure increases in rumination following that task^22,23,32–38^. All three of these methods have their own merits and drawbacks. While the investigation of spontaneous rumination might be the most valid, the use of experimental procedures allows causal inferences and an easier approach to differentiate state and trait aspects of rumination. However, experimental rumination induction paradigms might e.g. induce artificial neural activity that could be mistakenly attributed to the process of rumination. Therefore, with respect to the analysis of the neuronal correlates of rumination, it might be necessary to assess rumination as well as trait and state aspects during spontaneous and induced rumination to gain a full and conclusive picture of the process. On a neuronal level, depression generally is associated with reduced activity in the cognitive control network (CCN) during task performance^39^ and at rest^40^. Further, in MDD increased activity during resting state has been observed in the putamen and anterior cingulate on a meta-analytical level^40^. In line with this, during indirect induction of rumination by social stress, our group showed that low activity in the CCN during a stress task is related to increased state rumination following the stress task^22^. In contrast, studies using direct induction methods often find increased activity. For example, Burkhouse et al. (2016) observed increased activity within areas of the default mode network (DMN) but also in somatosensory areas during a rumination induction^26^. Further, Cooney et al. (2010) found increased activity within the dorsolateral prefrontal cortex (DLPFC), orbitofrontal cortex and subgenual anterior cingulate cortex^15^. In line with this, an fMRI task on self-criticism—a cognitive style associated with rumination^10,41^—was associated with increased activity within the lateral PFC^42^. A recent meta-analysis by Zhou et al. (2020) comparing several experimental studies using different direct paradigms to induce rumination, or self-referential thought, showed that these processes activate large portions of the DMN but also areas of the fronto-parietal network^19^.

In the current study, we investigated the qualitative nature of rumination in depressed patients and healthy controls (HC) and measured the neuronal correlates during spontaneous rumination and induced rumination by implementing an induction method that has been used by Berman et al. (2014) in an fMRI approach. In our study, we investigated the qualitative nature of rumination in MDD and HC additionally to the induction of rumination, as the process of rumination might not only differ between these groups quantitatively but also qualitatively. Given the clinical importance of the ruminative process and the heterogeneous findings on a neural level, such qualitative data is important for the interpretation of rumination induction paradigms. The induction of depressive rumination included two blocks of 180 s length during which subjects were instructed to think about two specific situations that had triggered rumination in the past. We used this direct induction method to compare the rumination levels at rest and during direct induction. To allow the investigation of neuronal activity during resting state and rumination induction, we investigated the amplitude of low frequency fluctuations (ALFF). Zang et al. (2007) were the first to analyze distinct alterations in ADHD patients by using ALFF^43^. Via Fast Fourier Transform, voxel-wise power spectra for frequencies between 0.01–0.08 Hz were obtained. ALFF is then implemented by calculating the square root of these frequencies and averaging it across the frequency range. Therefore, the ALFF index indicates the average amplitude of the signal in an investigated time-window. In comparison to classical block designs, the index can be applied without computing event-related averages and is therefore able to reflect a measure of cortical activity at resting state and during investigations of longer time periods that are suboptimal for classical averaging. Applying this method to the context of depression and rumination, Jing et al. (2013) showed an increased mean ALFF in the right medial frontal gyrus and a decreased mean ALFF in the right precuneus and left lingual gyrus for currently depressed patients relative to a healthy control group^44^. Guo and colleagues (2012) further utilized ALFF to show potential differences in treatment-resistant and treatment-responsive depression^45^. Interestingly, treatment-resistant patients show higher activations in the DMN, which could possibly serve as a biomarker for treatment indication in the future.

We hypothesized that depressed subjects would show higher levels of state rumination than healthy controls following the rumination induction as we expected a carry-over effect and that their qualitative description of rumination would differ from healthy controls (e.g. regarding its duration and controllability). The latter hypothesis was based on evidence that the emotional state of depressed subjects differs from that of healthy controls as well as their biographical background^46–49^ and therefore most likely their qualitative description of rumination might differ as well. Further, we aimed to find an increase in state rumination through the rumination induction as described by Berman et al. (2014). We expected increases in cortical activity of the areas of cognitive control (DLPFC, superior parietal lobule) and self-referential thinking (temporal lobule) during the rumination induction^19^. Finally, we expected that the increase in state rumination and related brain activity would be higher in the MDD group than in the HC group.

## Materials and methods

### Participants

26 depressed patients (MDD) and 26 healthy controls (HC) were recruited at the Department of Psychiatry and Psychotherapy of the University Hospital of Tübingen. Patients were contacted on the wards of the hospital and healthy controls were contacted via email and flyer. The used methods and procedures were in accordance with current guidelines of the World Medical Association Declaration of Helsinki and approved by the ethics committee at the University Hospital and University of Tübingen (287/2016B02). All participants gave written informed consent. All patients were diagnosed with current MDD by a clinical psychologist via a structured clinical interview for DSM-IV (SCID). In the same way, absence of any mental disorder was assessed in the HC. 69% (n = 18) of the clinical sample had a recurrent depressive disorder; 50% (n = 13) of all patients were diagnosed with a comorbid diagnosis including anxiety disorders (*n* = 8), eating disorders (*n* = 2), somatoform disorders (*n* = 1) and lifetime diagnosis of substance abuse (*n* = 2). 92.3% (n = 24) of all patients were, aside from the psychotherapeutic program, treated with antidepressant medication, including selective serotonin reuptake inhibitors (46.2%, n = 12), noradrenergic and specific serotonergic antidepressants (26.9%, n = 7), selective serotonin-norepinephrine reuptake inhibitors (19.2%, n = 5), norepinephrine-dopamine reuptake inhibitors (3.8%, n = 1) and tricyclic antidepressants (3.8%, n = 1), antipsychotics (46.2%, n = 12), and/or benzodiazepines (15.4%, n = 4).

The average Beck Depression Inventory (BDI-II) score was 32.26 (*SD* = 10.47) in the depressed group, which is equivalent to moderate depressive symptoms. The external assessment of depressive symptoms with the Montgomery-Åsberg Depression Rating Scale (MADRS) based on clinical ratings was 26.42 (*SD* = 8.53) indicating a moderate symptom severity. Further, patients showed a lower level of functioning as assessed by the Global Assessment of Functioning (GAF) and higher levels of trait rumination as assessed with the Rumination Response Scale (see Table 1). Patients showed a non-significant tendency to be older than the control subjects (${M}_{MDD}$ = 42 years, ${M}_{HC}$= 36 years, ${SD}_{MDD}$ = 11.70 years, ${SD}_{HC}$ = 13.25 years; d = 0.49). On average, the sample was 38.75 years old (*SD* = 12.76), was educated for 17.37 years (*SD* = 4.13) and 73% were female.

**Table 1:** Demographic variables of the depressed patients and healthy controls.

Variable	HC (*n* = 26)		MDD (*n* = 26)		*t/χ*^2^	*p*
	*M*	*SD*	*M*	*SD*		
Age (years)	35.65	13.25	41.85	11.70	*t* = 1.79	*p* < .1
Sex ratio (%female)	80% n = 21/5		65% n = 17/9		*χ*^2^ = .88	*p* > .1
Years of education	17.36	(4.49)	17.38	(3.87)	*t* < *1*	*p* > *.1*
Antidepressive medication (%)	0% n = 0/26		89% n = 24/2		*Χ*^*2*^ = 40.11	*p* < .001
MADRS	1.62	2.23	26.42	8.53	*t* = 14.35	*p* < .001
BDI-II	2.11	2.12	32.26	10.47	*t* = 14.40	*p* < .001
RRS	36.73	7.07	58.46	12.92	*t* = 7.52	*p* < .001
GAF	98.81	2.871	51.08	10.20	*t* = 22.97	*p* < .001

*MADRS* the Montgomery-Åsberg Depression Rating Scale, *BDI-II* Beck Depression Inventory II, *RRS* Rumination Response Scale, *GAF* Global Assessment of functioning.

### Procedure

**Figure 1:**
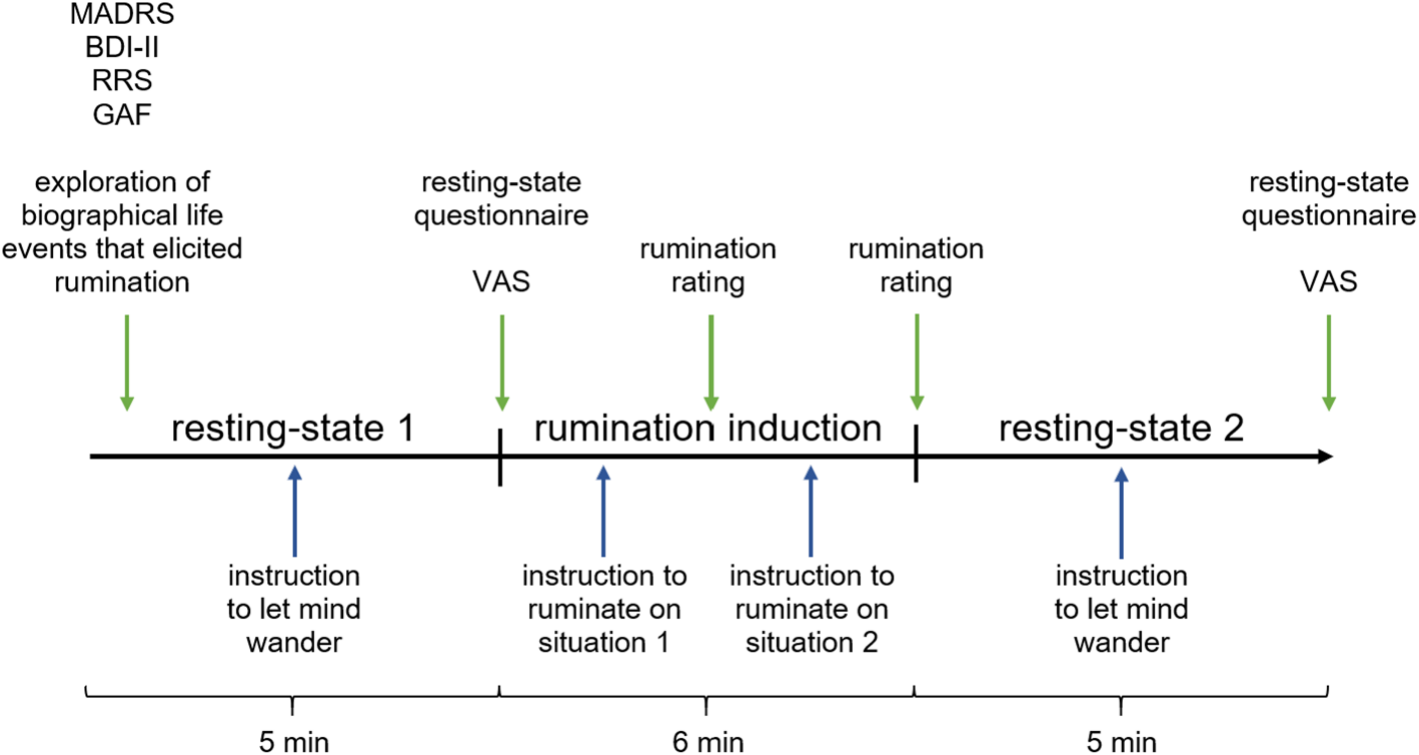
Time course of the conducted study.

At the beginning of the study, we assessed demographic and clinical variables (see Fig. 1). Aside from the questionnaires noted above, we assessed ruminative behavior of the participants in a semi-structured interview and the current emotional state with the Positive and Negative Affect Schedule (PANAS)^50^. In this interview participants were asked to describe their general ruminative behavior with respect to process, emotional, cognitive, as well as behavioral variables (see supplemental material and results). The interviewer directly rated the answers (Yes/No) of the participants on the answer sheet. We refer to this data as qualitative data as it mostly comprises answers on a nominal level. With respect to the rumination induction, we adapted a biographical rumination induction procedure as proposed by Berman et al. (2014). Biographical life events that had elicited rumination in the past were explored from each participant preceding the experimental procedure. Each biographical situation was briefly described, one to three cue words were selected and ratings were given for the emotional load of the situation, memory accessibility and personal actuality of the situation by the subject. Out of the four situations, the two with the highest scores in these ratings were selected. The experimental procedure was as follows: Each participant had a 5 min resting phase in which they were asked to sit still, close their eyes and let their mind wander. After the resting state measurement, subjects documented processes that had occurred during the measurement on an adapted version of the Amsterdam resting state questionnaire^51,52^ (ARSQ) and Visual Analogue Scale (VAS) used by our group^13,23,51^. From the ARSQ, we used the scales of discontinuity of mind, self, planning, comfort and added a scale on rumination (see appendix).

These questionnaires were followed by the rumination induction. The keywords of the situation were presented to the subjects, which were then asked to ruminate on the event. They were instructed to think about “how they got into the situation”, “what they have done wrong” and “the consequences of the situation”. After the instruction and presentation of the keywords, subjects were asked to close their eyes and to ruminate on the situation for 3 min. In total, each subject ruminated on two situations for 6 min. After the rumination induction subjects were asked to rate how well they remembered the situation, how well they could stay in the situation and to which extent they ruminated on a scale from 1 to 10. Finally, a second resting state measurement equal to the first resting phase was conducted. At the end of the resting state measurement, the VAS, ARSQ and PANAS were assessed a second time.

#### Questionnaires

In the study, several questionnaires and measures have been used that will be explained in the following. Means, standard deviations and correlations of the measures can be seen in supplementary tables S1 and S2.

#### MADRS

In order to screen depression symptom severity, we used the Montgomery–Åsberg Depression Rating Scale (MADRS)^53^. This measure consists of 10 items, with each item yielding a score of 0 to 6 and summing up to a total score ranging from 0–60. Due to its efficiency and high inter-rater reliability of r = 0.76, this measure is optimized for rapid clinical use^53,54^.

#### BDI

As another tool to assess depression symptom severity, we used the self-report questionnaire Beck Depression Inventory II^55^. Regarding the previous two weeks, the occurrence of 21 symptoms is rated on a 4-point Likert Scale and symptom severity is assessed as a total score ranging from 0–63. Investigating psychometric properties across different populations and languages, respectively, Wang et al. (2013) could observe overall high internal consistencies (Cronbach’s α approx. 0.9) as well as high test-retest reliabilities (mean interval of 2 weeks; r = 0.7–0.9)^56^. Especially important for our study, the German version has been shown to satisfactorily differentiate between depressed patients and healthy controls^57^ and is further considered a good screening tool for Major Depressive Disorder^58^.

#### GAF

The Global Assessment of Functioning is a rating scale of the overall social, occupational, and psychological functioning of an individual. Resulting total scores range from 100 (extremely high functioning) to 1 (severely impaired). This clinician-administered scale was also part of the first interview. Previous studies investigating the psychometric properties of the GAF found medium to high inter-rater reliabilities (intraclass correlation coefficients range: ICC = 0.5–0.81)^59,60^.

#### RRS

We measured ruminative responses using the Ruminative Response Scale. This self-report questionnaire assesses ruminative processes as a trait using a total of 22 items which are rated on a 4-point Likert scale from "hardly ever" to "almost always". Two subscales can be distinguished, brooding and reflective pondering, which could range from 22–88. Cronbach´s ɑ of the RRS has been shown to be good α_C_ = 0.88–0.92^9,61,62^.

#### PANAS

Participants rated the extent to which they experienced several emotions at the moment using items of the Positive and Negative Affect Schedule^63^. The 20 items are assessed using 5-point Likert scales that range from 1 (“very slightly”) to 5 (“extremely”) and can be divided into two subscales: positive affect (PA) and negative affect (NA), which have acceptable internal consistencies in clinical and non-clinical samples^50,64^ of ɑ = 0.85–0.86 for NA and ɑ = 0.84–0.89 for PA. Further, Watson and colleagues (1988) found high test-retest reliabilities of both, PA and NA (mean interval of 8 weeks; PA: r = 0.68, NA: r = 0.71)^63^.

#### ARSQ

The Amsterdam Resting-State Questionnaire consists of several scales measuring thoughts and feelings that can occur during resting-state and follows a 5-point Likert scale^51,52^. From the original scales, we included the scale of “Discontinuity of Mind” (3 Items), “Self” (3 Items), “Comfort” (3 Items), and “Planning” (3 Items). Additionally to the items of the original ARSQ, we added items from the RRS (see supplemental material) to assess a state rumination scale. This scale had a test–retest reliability between the resting state measurements of r_tt_ = 0.84 and a Cronbachs Alpha of α_C_ = 0.95.

#### VAS

The VAS consisted of 3 items from 0 to 100% to measure rumination and 2 items to assess mind-wandering (see appendix). Test–retest reliability between resting state 1 and resting state 2 for the rumination scale was r_tt_ = 0.84 (α_C_ = 0.87) and for the mind-wandering scale r_tt_ = 0.42 (α_C_ = 0.5).

### fNIRS

**Figure 2:**
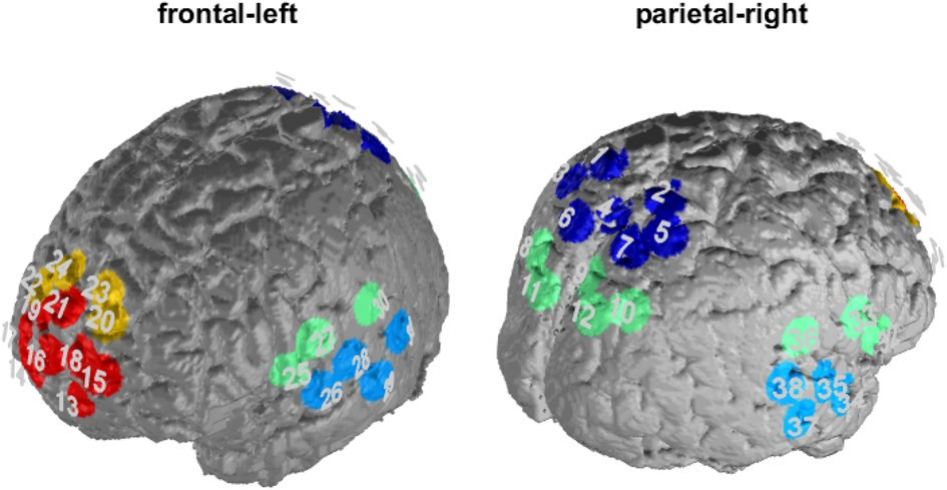
Channel positions and regions of interest (ROI). Red = frontopolar channels, Yellow = dorsolateral prefrontal cortex, light blue = temporal areas, dark blue = somatosensory association cortex.

The optical imaging method of functional near infrared spectroscopy (fNIRS) was used to measure changes of oxygenated (O_2_Hb) and deoxygenated haemoglobin (HHb). fNIRS has a relatively high temporal resolution and is capable of measuring cortical changes in O_2_Hb and HHb^65,66^. We used a continuous wave, multichannel NIRS system (ETG-4000 Optical Topography System; Hitachi Medical Co., Japan) with a temporal resolution of 10 Hz. To measure parts of the CCN and DMN, we placed an optode-system with 4 probesets: One frontal probeset was placed with reference position Fpz, a parietal probeset with reference to Pz and two temporal probesets with reference to T3 and T4 according to the 10–20 system^67^ (see Table 2, Fig. 2).

**Table 2:** fNIRS channels and related brain areas (estimates are based on a neuro-navigational measurement of an exemplary volunteer).

Brain area	Channels
Orbitofrontal cortex	14
Dorsolateral prefrontal cortex	22 20 23 24
Frontopolar area	13 15 16 17 18 19 21
Middle temporal gyrus	26 29 34 37
Retrosubicular area	32 33 36
Superior temporal gyrus	28 31 35 38
Subcentral area	27
Primary somatosensory cortex	30
Somatosensory association cortex	1 2 3 4 5 6 7
V3	8 9 10 11 12
Premotor and supplementary motor cortex	25

### Data analysis

#### Preprocessing

Data was processed and analyzed using MATLAB R2017a (MathWorks Inc, Natick, USA) and PASW (Version 24). After O_2_Hb and HHb signals were computed by means of a modified Beer-Lambert Law, data preprocessing included: correction of movement artefacts by the TDDR algorithm^68^, bandpass filtering (0.1–0.01 Hz), correction of O_2_Hb data by the correlation-based signal improvement algorithm^69^, visual inspection and single channel interpolation in case of artefacts and a global signal reduction with a spatial gaussian kernel filter^70^ with a standard deviation of σ = 38. After preprocessing, the amplitude of low frequency fluctuations was computed^44^. We chose not to assess the average hemodynamic response during the 3 min rumination induction due to the implemented filters. The application of a high-pass filter at 0.01 Hz is critical for the removal of low frequency drifts of the signal. However, such a filter also ensures an activity near zero over the course of 3 min as the filter reduces the low-frequency activity in the time window (one oscillation in 3 min would imply an effect in a frequency lower than 0.005 Hz). A solution for the assessment of activity during the 3 min offers the assessment of the amplitude of low frequency fluctuations. We assessed the power of the frequency spectrum of 0.01 to 0.1 Hz by the *pwelch* function of MATLAB (Welch’s power spectral density estimate) with 30 s time windows. Afterwards, we computed the average amplitude of the fluctuations as the mean of the square root of the power spectrum for each frequency bin. The resulting metric offers the average amplitude within the frequency range without addressing positive or negative values that would result in a near zero hemodynamic response when computing the simple average of the signal. Note that we did not standardize ALFF values as unlike in fMRI data, fNIRS data provided non-arbitrary values. However, recent developments in amplitude fluctuation research suggest various parameters and algorithms to assess signal fluctuations. A recent publication suggests that the Percent Amplitude of Fluctuation (PerAF) might be a more robust parameter, as it accounts for the arbitrary units of fMRI data^71^. To compare the ALFF data, we additionally calculated and analyzed the PerAF.

Figures were created using ggplot2 in R and brain maps using self-written code in MATLAB 2017a. With respect to the brain maps, differences in Cohen's d were mapped on a template brain at the respective channel coordinates. The brain surface voxels between the channels were interpolated using Gaussian radial basis functions^65^. Cohen's d was computed as:

$$d = \frac{{x}_{1}-{x}_{2}}{\sqrt{\frac{{VAR}_{x1}+{VAR}_{x2}}{2}}}$$

### Statistical analysis

We analyzed the interview regarding ruminative behavior, rumination during the experiment and ALFF assessed via fNIRS during resting state and rumination induction. Differences in general ruminative behavior as assessed by the semi-structured interview were analyzed by separate χ^2^-tests and a single *t*-test between healthy controls and depressed patients. Further, we conducted a discriminant analysis to identify the most important dimensions of rumination to investigate potential differences in depressed patients and healthy controls. Behavioral ratings and questionnaire data were analyzed with repeated measurement MANOVAs. With respect to fNIRS data, we conducted a repeated measurement ANOVA with the factors condition (resting state 1 vs. rumination induction vs. resting state 2) and region of interest (DLPFC, frontopolar, temporal left, temporal right, SAC). By using the Benjamini–Hochberg procedure, we corrected for multiple comparisons.

## Results

### Qualitative data

The analysis of the semi-structured interview of ruminative behavior resulted in differences between MDD patients and healthy controls in nearly all variables assessed (see Table 3). Depressed subjects reported less control over ruminative behavior (uncontrollability = 93% vs. 15%), higher impairment of rumination (impairment = 89% vs. 11%), longer durations (approx. 3 h vs. 15 min/day), increased dwelling on thoughts (100% vs. 62%) and a higher focus on the past (68% vs. 27%) (see Table 3). Both groups did not differ in how far the topic of their rumination was related to relationship (46% vs. 57%) and job (23% vs. 28%) issues. However, MDD patients reported a higher personal relevance (88% vs. 64%) and more ruminations on personal failures (0% vs 46%). Rumination-related emotions such as guilt, shame or sadness were more present in depressed subjects (50–65%) than in healthy controls (4–8%). Further, MDD patients reported more hopelessness during rumination (71% vs. 8%) and less goal-directed processing (4% vs. 54%), but no differences were found with respect to optimism about the future (25% vs. 50%) and concreteness of ruminations (57% vs. 64%). Lastly, counterfactual thinking during rumination was more present in MDD patients (90% vs. 27%) and ruminations were less often followed by actions compared to healthy controls (10% vs. 54%).

**Table 3:** Results of the qualitative interview of depressed subjects and healthy controls on their ruminative tendencies. Significant p-values and effect sizes are depicted in bold for reasons of clarity.

	HC	MDD	*t/χ*^*2*^	*p*	*OR/d*
Process: dwell on thoughts	62%	100%	*χ*^*2*^ = 13.22	***p < .001***	***OR = 1.62***
Process: uncontrollability	15%	93%	*χ*^*2*^ = 32.77	***p < .001***	***OR = 6.2***
Process: focus on past	27%	68%	*χ*^*2*^ = 7.74	***p < .01***	***OR = 2.52***
Process: duration (hours/day)	0.23	2.8	*t* = 6.41	***p < .001***	***d = 1.77***
Process: impairment	11%	89%	*χ*^*2*^ = 32.64	***p < .001***	***OR = 8.09***
Thematic: personal relevance rated from 0 to 100%	64%	88%	*t* = 3.05	***p < .01***	***d = 0.8***
Thematic: job	23%	28%	*χ*^*2*^ = 0.21	*p* > .1	*OR* = 1.20
Thematic: relationships	46%	57%	*χ*^*2*^ = 0.21	*p* > .1	*OR* = 1.23
Thematic: personal failure	0%	46%	*χ*^*2*^ = 15.90	***p < .001***	***OR = 25.14***^**a**^
Emotion: guilt	8%	57%	*χ*^*2*^ = 14.48	***p < .001***	***OR = 7.12***
Emotion: shame	4%	50%	*χ*^*2*^ = 14.31	***p < .001***	***OR = 12.5***
Emotion: sadness	8%	65%	*χ*^*2*^ = 18.52	***p < .001***	***OR = 8.13***
Cognition: hopelessness	8%	71%	*χ*^*2*^ = 22.68	***p < .001***	***OR = 8.87***
Cognition: optimism in future	50%	25%	*χ*^*2*^ = 4.81	*p* > .1	*OR* = 0.5
Cognition: solution	54%	4%	*χ*^*2*^ = 12.41	***p < .001***	***OR = 0.07***
Cognition concreteness	57%	64%	*χ*^*2*^ = 0.25	*p* > .1	*OR* = 1.12
Cognition: counterfactual	27%	89%	*χ*^*2*^ = 20.95	***p < .001***	***OR = 3.30***
Behavior: action	54%	10%	*χ*^*2*^ = 11.63	***p < .001***	***OR = 0.185***

All p-values are calculated as Fisher's exact tests.

^a^Computed with Haldane-Anscombe correction.

Finally, a discriminant analysis using a stepwise approach indicated that 94% of subjects were correctly classified as patients or healthy controls by taking the following variables into account: process-uncontrollability (*F*(1, 51) = 76.898, *p* < 0.001), cognition-solution (*F*(2, 50) = 56.202, *p* < 0.001), emotion-sadness (*F*(3, 49) = 45.867, *p* < 0.001) and process-dwelling on thoughts (*F*(4, 48) = 37.374, *p* < 0.001). Note that a cross validation using the leaving-one-out method resulted in an 88.9% correct classification using these variables.

### Behavioral data

For behavioral measures, we analyzed the ratings of the rumination induction paradigm and state-measures after resting state measurements with MANOVAs.

The analysis of the ratings during the rumination induction indicated a significant main effect of group (MDD vs. HC: *F*(3, 47) = 5.711,* p* < 0.01, Wilks *Λ* = 0.73, ${\eta }_{p}^{2}$ = 0.27) with univariate analysis revealing no differences in remembering (M_MDD_ = 7.5, SD = 1.36, M_HC_ = 7.8, SD = 1.66) or staying (M_MDD_ = 6.88, SD = 1.77, M_HC_ = 6.67, SD = 1.65) in the situation but the amount of ruminations (*F*(1, 49) = 15.298, *p* < 0.001,${\eta }_{p}^{2}$ = 0.24) with patients showing more ruminations than controls (M_MDD_ = 7.0, SD = 1.55, M_HC_ = 4.85, SD = 2.29). No effects were found with respect to an interaction of group by measurement point.

Changes from the first to the second resting state measurement were assessed with a repeated measurement MANOVA on the dependent variables of the PANAS, ARSQ and VAS. The results showed a significant main effect for group (MDD vs. HC: *F*(9, 40) = 9.682, *p* < 0.001, Wilks *Λ* = 0.319, ${\eta }_{p}^{2}$ = 0.68) and measurement point (pre vs. post: *F*(9, 40) = 3.746, *p* < 0.01, Wilks *Λ* = 0.588, ${\eta }_{p}^{2}$ = 0.46). Univariate analysis of the main effect of measurement point indicated a significant decrease in ARSQ comfort (*F*(1, 48) = 11.339, *p* < 0.01, ${\eta }_{p}^{2}$ = 0.19, M_Pre_ = 3.0, SD = 1.18, M_Post_ = 2.56, SD = 0.95) and positive affect from pre to post (*F*(1, 48) = 10.225, *p* < 0.01, ${\eta }_{p}^{2}$ = 0.17, M_Pre_ = 26.34, SD = 7.95, M_Post_ = 24.14, SD = 8.37) and an increase in the VAS rumination scale (*F*(1, 48) = 18.851, *p* < 0.001, ${\eta }_{p}^{2}$ = 0.28, M_Pre_ = 2.3, SD = 2.51, M_Post_ = 3.17, SD = 2.5). Note that the ARSQ rumination scale showed a tendency towards an increase in state rumination before, but not after correction for multiple comparisons (*F*(1, 48) = 4.609, *p* < 0.05, ${\eta }_{p}^{2}$ = 0.09). Univariate analyses of the main effect of group indicated significant differences between the groups in all scales but ARSQ planning. The MDD group showed higher ARSQ measured rumination (*F*(1, 48) = 81.941, *p* < 0.001, ${\eta }_{p}^{2}$ = 0.62, M_HC_ = 1.09, SD = 0.21, M_MDD_ = 2.4, SD = 0.73), higher self-related thoughts (*F*(1, 48) = 34.656,* p* < 0.001, ${\eta }_{p}^{2}$ = 0.41, M_MDD_ = 3.17, SD = 0.66, M_HC_ = 2.19, SD = 0.51), lower comfort (*F*(1, 48) = 23.846, *p* < 0.001, ${\eta }_{p}^{2}$ = 0.33, M_MDD_ = 2.26, SD = 0.76, M_HC_ = 3.33, SD = 0.80), higher discontinuity of mind (*F*(1, 48) = 25.590, *p* < 0.001, ${\eta }_{p}^{2}$ = 0.34, M_MDD_ = 2.95, SD = 0.80, M_HC_ = 1.96, SD = 0.57), higher VAS rumination (*F*(1, 48) = 36.666, *p* < 0.001, ${\eta }_{p}^{2}$ = 0.43, M_MDD_ = 4.34, SD = 2.48, M_HC_ = 1.11, SD = 0.89), lower VAS mind wandering (*F*(1, 48) = 11.685, *p* < 0.001, ${\eta }_{p}^{2}$ = 0.19, M_MDD_ = 5.84, SD = 1.59, M_HC_ = 7.45, SD = 1.76), lower positive affect (*F*(1, 48) = 20.998, *p* < 0.001, ${\eta }_{p}^{2}$ = 0.30, M_MDD_ = 20.98, SD = 6.07, M_HC_ = 29.5, SD = 7.03) and higher negative affect (*F*(1, 48) = 49.912, *p* < 0.001, ${\eta }_{p}^{2}$ = 0.51, M_MDD_ = 25.0, SD = 9.42, M_HC_ = 11.6, SD = 1.03), when compared to the HC group.

### Neurophysiological data

To investigate the effect of depression status on ALFF during the rumination induction, we conducted a three-way repeated measures ANOVA with the within-subject factors ROI (frontopolar, DLPFC, temporal-left, temporal-right, SAC) and condition (resting state 1, rumination induction, resting state 2) and the between-subjects factor group (depressed, non-depressed).

**Figure 3:**
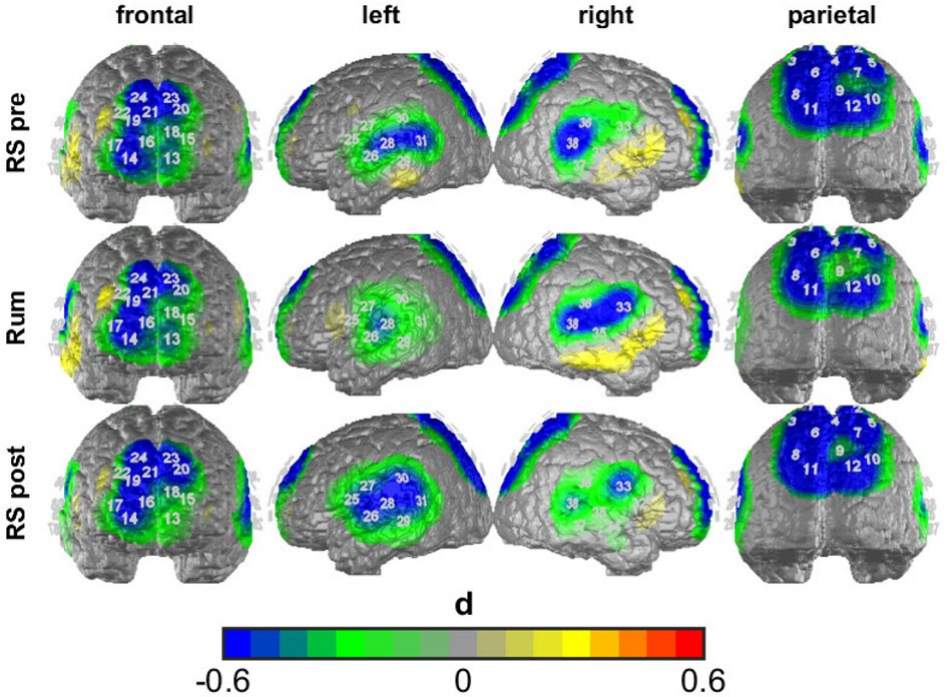
Differences between the MDD group and the HC group in ALFF during the different experimental conditions. Cold colors indicate higher activation in the HC than in the MDD, warm colors indicate higher activation in the MDD than in the HC.

**Figure 4:**
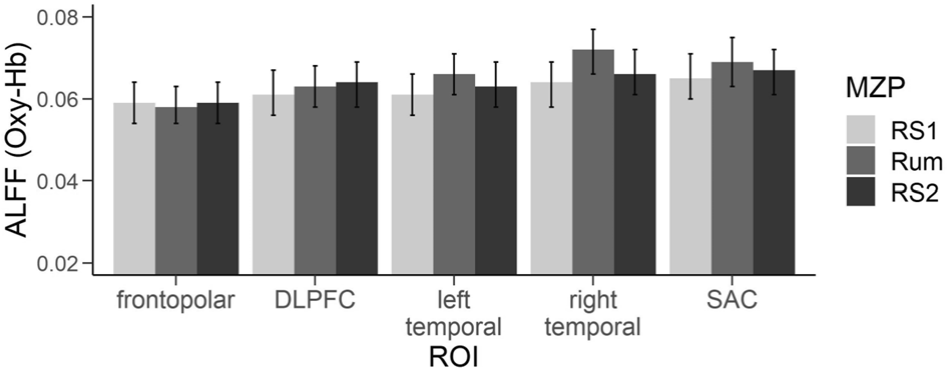
Interaction of ROI and condition in ALFF (Oxy-Hb) with error bars indicating standard errors. *RS* resting state, *Rum* Rumination induction, *ROI* Region of interest, *ALFF* Amplitude of Low Frequency Fluctuations. The scaling of the y-axis has been set to a minimum of .02 which is equal to the smallest value we measured in ALFF in this sample.

Our analysis indicated a significant main effect of group (*F*(1, 49) = 7.450, *p* < 0.01, ${\eta }_{p}^{2}$ = 0.29), ROI (*F*(4, 196) = 3.715, *p* < 0.05, ${\eta }_{p}^{2}$ = 0.07), condition (*F*(2, 98) = 3.929, *p* < 0.05, ${\eta }_{p}^{2}$ = 0.07) and an interaction of ROI by condition (*F*(8, 392) = 2.507, *p* < 0.05, ${\eta }_{p}^{2}$ = 0.05). The main effect of group indicated overall lower brain activity in the MDD group in comparison to the HC group (see Fig. 3). Polynomial contrasts of the effect of condition showed a significant quadratic relationship (*F*(1, 49) = 6.847, *p* < 0.05, ${\eta }_{p}^{2}$ = 0.12) with an increase in activity from the first resting state to the rumination induction and a following decrease from the rumination induction to the second resting state (see Fig. 4).

Post-hoc tests regarding the interaction effect of ROI by condition were performed on each ROI separately, as direct comparisons between different ROIs might be difficult due to the differential path lengths of the NIR light. After correcting for multiple comparisons, only post-hoc comparisons in the right temporal cortex revealed a significant quadratic relationship in the before-mentioned direction (*F*(1, 49) = 9.488, *p* < 0.01, ${\eta }_{p}^{2}$ = 0.16). Note that tendencies were also found in the left temporal cortex (*F*(1, 49) = 4.590, *p* < 0.05, ${\eta }_{p}^{2}$ = 0.86) and SAC (*F*(1, 49) = 5.489, *p* < 0.05, ${\eta }_{p}^{2}$ = 0.10) before, but not after the correction by Benjamini-Hochberg.

### PerAF analysis

**Figure 5:**
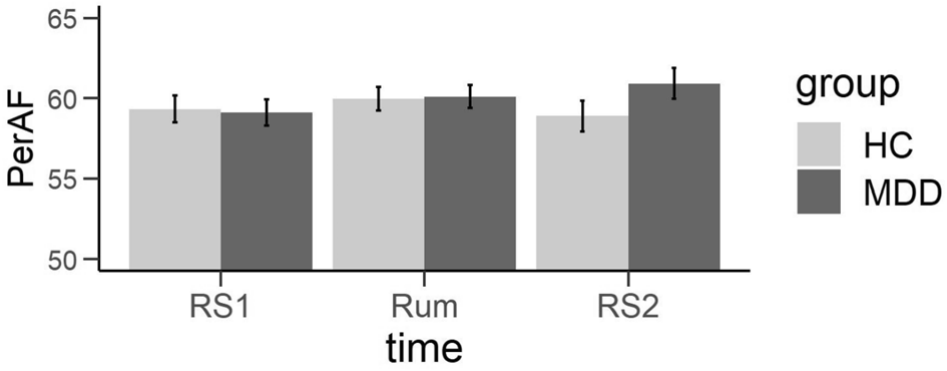
Interaction of condition by group in PerAF. *PerAF* Percent amplitude of fluctuation, *RS1* resting state, *RUM* rumination induction, *HC* healthy controls, *MDD* depressed patients.

With respect to the PerAF analysis, our repeated measurement ANOVA showed a significant main effect of ROI (F(4, 196) = 8.393, p < 0.001, ${\eta }_{p}^{2}$ = 0.15) and an interaction of condition by group (F(2, 98) = 5.321, p < 0.01, ${\eta }_{p}^{2}$ = 0.10). Note that a marginally significant main effect of condition (F(2, 98) = 2.807, p < 0.1, ${\eta }_{p}^{2}$ = 0.05) was present as well. Post-hoc analysis of the interaction of condition by group revealed significant differences between the diagnostic groups in the linear contrast of condition (F(1, 49) = 8.344, p < 0.01, ${\eta }_{p}^{2}$ = 0.15): While the MDD group showed a linear increase from the first to the second resting state in PerAF values, the HCs showed no such linear increase but a tendency for a quadratic relationship like in the ALFF analysis (see Fig. 5).

### Exploratory analysis

Finally, we explored correlations between trait rumination as measured with the RRS, state rumination (VAS and ARSQ) and fNIRS activity to explore between-subject correlations between cortical activation and rumination. We did not find significant correlations with any rumination scale (all *p* > 0.05). Marginal tendencies towards negative associations between state rumination as measured with the ARSQ were found in the SAC, DLPFC and left temporal ROI (*r*(52) = − 0.23 to − 0.30, *p* < 0.1). In the same way, PerAF values only showed a tendency towards positive correlations between measured state rumination using the ARSQ and amplitude fluctuations during the second resting state measurement in the SAC (*r*(52) = 0.29, *p* < 0.05).

### Confounding variables

We further explored if the age of the participants significantly influenced the results by reanalyzing our results using age as a covariate. Our analysis showed that the behavioral increase in state rumination—as assessed with VAS and ARSQ—as well as the group differences between patients and healthy controls was still significant after using age as a covariate. Age showed to be a significant predictor in the analysis of ALFF data (F(1,47) = 6.479, p < 0.05,${\eta }_{p}^{2}$ = 0.13) and was negatively correlated with ALFF in the DLPFC and SAC (r(50) = −.41 to − 0.49, p < 0.01). The main effects of patient group and condition were still significant after including age as a covariate. In the same way, the interaction of condition by group remained significant in the analysis of PerAF values.

## Discussion

The current study aimed to investigate the effects of depressive rumination on ALFF assessed with fNIRS during resting state and a rumination induction paradigm. To this end, we recruited a sample of patients with MDD and healthy controls, assessed measures of state and trait rumination and measured ALFF during a rumination induction paradigm and resting states.

Our qualitative and quantitative data revealed that depressed subjects showed a typical pattern of increased trait and state rumination. Further, in our qualitative interview we were able to find significant deviations in the quality of ruminative behavior between depressed subjects and healthy controls in nearly all assessed dimensions of rumination. 94% of the subjects were discriminable into depressed patients and healthy controls by the variables controllability, dwelling on thoughts, emotional sadness and lack in solution finding during rumination. These qualitative descriptions imply that although ruminative cognitions are dimensional constructs that can be found in all humans, the quality of ruminations in depressed subjects differs in important aspects such as controllability, impairment, affective tone and emotional thematic content. As such, the investigation of rumination in healthy participants might be advantageous in terms of confounding factors such as medication but yields results that are not comparable to ruminative processes in clinical samples.

The MDD group reported higher levels of state rumination at both resting state measurements and during the rumination induction. Although we did not find an interaction of group by condition, we observed an increase in state-measures of rumination from the first to the second resting state measurement, regardless of group. However, we did not find an interaction effect as in other studies^22^, which somehow limits the powerfulness and therefore usefulness of the current paradigm. Depressed subjects reported higher rumination during the rumination induction; however, their ruminations during the following resting state measurement were not differently increased related to their baseline ruminations, indicating a decline compared to the healthy controls. From other investigations of our group using social stress paradigms^22,23^, we expected the rumination induction to show carry-over effects in the MDD group.

Similarly, on a neuronal level, we observed two separate effects: A main effect of group indicating reduced ALFF in the depressed subjects and a main effect of condition (as well as an interaction of condition by ROI) indicating an increase in ALFF during the rumination induction in general and especially in the right temporal cortex. Interestingly, like in the study of Bermann et al. (2014) on relations between functional connectivity and rumination, our analysis leaves us with contradictory findings. On the one hand, depressed subjects showed a well-known pattern of hypoactivity^39,40^, as indicated by the main effect of group on ALFF data, and increased behavioral indices of trait and state rumination. On the other hand, rumination induction was related to an increase in ALFF in temporal and parietal regions and an increase in state rumination, regardless of group. These results could be interpreted in two possible ways. First, the generally reduced ALFF in depressed subjects might be the “true effect” reflecting depressive status and depressive rumination, while the induction-related increase in activity is a reflection of increased cognitive load due to the task. Second, the reduced ALFF in depression might not be a correlate of depressive rumination and the increase in ALFF during the rumination induction is the correlate of state rumination. Although the current study will not be able to disentangle this question completely, the first interpretation might be more likely to be the right one. We found increased activity during the rumination induction, which then declined during the second resting state measurement, although behavioral measures of state rumination increased during this resting state measurement. This assumption may be supported by the tendencies towards negative associations between cortical oxygenation and state rumination measures. However, it is important to note that the generally decreased ALFF in the depressed group might indeed be a result of allostatic changes in brain functioning that can be mediated by rumination. For example, the perseverative cognition hypothesis states that rumination prolongs the duration of high-stress states in high ruminators. Them being exposed to prolonged stress responses, this might lead to chronic stress and allostatic changes^72,73^. In line with this, we observed no specific association between changes in state rumination and changes in functional connectivity in high trait ruminators through a stress induction paradigm, but reduced prefrontal activity in high ruminators during stress, which mediated the relationship between trait rumination and increased state rumination after stress^22,23^. The result of decreased ALFF in MDD is further in line with other studies investigating ALFF in depression, although results on ALFF differences in patients with MDD vary between ROI in the literature^44,45,74^. Generally, depression has been shown to be related to hypoactivity especially in the prefrontal cortex on a meta-analytic level (Zhang et al., 2015). In previous studies, the induction of rumination has been associated with increased activity within areas of the DMN and CCN^15,26,42,75^. In line with this, we observed a general increase in ALFF during the rumination induction in the right temporal cortex and superior parietal lobule. However, such induction paradigms themselves might induce artificial activity, e.g. through cognitive load^76^. With respect to our results, this interpretation might be very likely as imagination paradigms per se are related to activity in prefrontal, temporal and parietal cortex areas^77^. Alternatively, it could be argued that physiological arousal effects (e.g. changes in blood pressure) might have contributed to the task-related changes in ALFF. However, we used several correction methods such as TDDR, CBSI and PCA-based correction to account for this type of confounding effect. On the other hand, as the effect of condition is not specific to the clinical group and the effect of group is independent of the condition (resting state 2 vs. rumination induction vs. resting state 2), the interpretation of general physiological effects cannot be ruled out completely. For example, depressed subjects could show aberrant respiration which would have systematic effects on the cortical hemodynamic response^78^. Interestingly, the analysis of the PerAF values resulted in some different results than the ALFF analysis. Contrarily, we observed a significant interaction of group by condition which was driven by a linear increase in PerAF from the first to the second resting state measurement in patients with MDD but not the HC group. Noteworthy, unlike in ALFF analysis, no main effect of group was observed, which is probably due to the standardization procedure of the PerAF algorithm that centers the data within each subject and therefore limits between-group variations. Importantly, this procedure is very necessary in arbitrary fMRI data, but might not be needed in case of fNIRS. Although the interaction of group by condition showed a selective increase in PerAF in the MDD group as hypothesized, the data was not significantly associated with state rumination after correction for multiple comparisons, which limits the interpretation of this increase. The interpretation of this effect is further limited as no interaction of group by condition was found in the behavioral data of state rumination. However, like the general condition effect for ALFF parameter, the linear increase of PerAF in the MDD group could be interpreted as an increase due to the rumination induction.

It is important to note that none of the paradigms for the investigation of depressive rumination is perfect in design. For example, the study of spontaneous rumination during resting state might seem to be the purest measurement of the neuronal correlates of rumination, however, not all subjects might ruminate during resting state and such investigations would require the assessment of state rumination on a behavioral level, which is often not performed. Further, these studies are merely correlational and, therefore, do not offer the opportunity of drawing causal attributions. Lately, indirect induction methods have been used for provoking rumination. In this context, social stress tests such as the Trier Social Stress Test^79^, adapted versions or similar paradigms are often used. Indeed, most studies using this approach were able to show increased rumination after social stress^22,33–36,38,80^. However, the stress response itself might trigger neuronal changes (e.g. stress adaptive processes), which could be confounded with psychometric measures of rumination. The classical approach of inducing rumination is to present the participants a prompt of some kind of trigger (e.g. thinking about personal shortcomings; Berman et al., 2014). Although this approach has been used widely in psychometric and neuronal settings, the induction might corrupt the ruminative process while an increase in rumination in psychometric measures is found. For example, one induction method includes thinking about specific topics (e.g. why you feel the way you feel right now) and measures depressive rumination after the induction by using exactly this item (e.g. during the last 5 min, did you think about your feelings and why you feel the way you feel right now). Although thinking about self-referential topics is a typical content of depressive rumination, other process-related aspects of rumination (e.g. the rather involuntary, repetitive and uncontrollable nature of depressive rumination) might not be captured by such induction methods. Further, these methods also might induce artificial neuronal activity due to cognitive load, which is then attributed to depressive rumination. However, on the other hand, this paradigm represents the most direct induction of rumination.

Some important limitations have to be considered with respect to our results. First, despite the advantages of fNIRS, the depth resolution of the method is limited to the upper 2–3 cm^66^. Therefore, we were not able to investigate subcortical changes in ALFF. Further, most of the depressed subjects were medicated, which could influence ALFF during resting states and rumination induction. However, state rumination was still increased in this sample and therefore neuronal correlates of this cognitive process should be measurable. As already reported, we did not observe carry-over effects of the rumination induction, which to some extent limits the power of the used induction method. One possible explanation could lie in the pharmacotherapeutic treatment of the patients. As nearly all patients were treated with antidepressant medication, we could not investigate the potential role of medication. However, the investigation of untreated patients yields some difficult ethical problems. Therefore, an induction paradigm needs to be sensitive also in cases of treated patients. In a current study using a stress paradigm for the induction of rumination, we observed differential effects independent from treatment status. Lastly, the analyzed sample size is rather small and the results should therefore be interpreted with caution. However, this first pilot data showed highly significant and important first starting points for the conceptualization of rumination induction paradigms.

In conclusion, the current study found evidence for a successful rumination induction through a direct induction paradigm; however, the induction seems to be limited as the rumination induction was not specific to depressed subjects. Further, on a neuronal level contradictory evidence was found with a well-known pattern of hypoactivity in depressed subjects and an increase in temporo-parietal activity during rumination induction which might be attributed to cognitive load. In future studies it will be important to investigate different paradigms in the same clinical and non-clinical samples as well as in samples at risk for depression in longitudinal investigations. Like that, it will be possible to disentangle the effects of trait and state rumination as well as allostatic changes on a neuronal level. Further, it should be investigated in greater detail if imaginary paradigms induce carry-over effects and are valid in terms of rumination in everyday life (e.g. assessed though momentary assessment) as otherwise the powerfulness of such paradigms might be limited.

## Supplementary Information

Supplementary Information

## Data Availability

The data of this study is available upon request from the first author.
