# Supplementary material for: Amplitude of low frequency fluctuations (ALFF) of spontaneous and induced rumination in major depression: An fNIRS study
Source: Sci Rep. 2020 Dec 9;10:21520. doi: 10.1038/s41598-020-78317-y (PMC7725822; doi:10.1038/s41598-020-78317-y)
Supplement: Supplementary file 1 — Supplementary Information [file 41598_2020_78317_MOESM1_ESM.docx]

**Supplemental Material: Amplitude of low frequency fluctuations (ALFF) of spontaneous and induced rumination in major depression: An fNIRS study**

David Rosenbaum^1^, Isabell Int-Veen^1^, Agnes Kroczek^1^, Paula Hilsendegen^1^, Kerstin Velten-Schurian^1^, Isabel Bihlmaier^1^, Andreas J. Fallgatter^1,2,3^, Ann-Christine Ehlis^1,2^

^1^ Department of Psychiatry and Psychotherapy, University Hospital of Tuebingen, Tuebingen, Germany

^2^ LEAD Graduate School & Research Network, University of Tübingen, Germany

^3^ German Center for Neurodegenerative Disorders, Tübingen, Germany

**Corresponding Author:**

David Rosenbaum

Calwerstraße 14

72076 Tübingen

Germany

email: david.rosenbaum@med.uni-tuebingen.de

Phone: 00497071 29-83609

ARSQ Items:

Comfort:

- I felt comfortable
- I felt relaxed
- I felt happy

Discontinuity of mind:

- I had busy thoughts
- I had rapidly switching thoughts
- I had difficulty holding on to my thoughts

Planning:

- I thought about things I need to do
- I thought about solving problems
- I thought about the future

Self

- I thought about my feelings
- I thought about my behavior
- I thought about myself

Added subscale on rumination was adapted from the RRS:

- I thought about all my shortcomings, failings, faults and mistakes.
- I thought about why I can’t handle things better.
- I thought about why I have problems other people don’t have.
- I thought about whereby I deserved my current life situation.
- I couldn’t leave my negative thoughts aside.
- I thought about past situations that I regret.
- I thought about all my problems and worries.

VAS scales for assessing processes during the resting-state comprised the following items:

Mind-wandering:

1. I felt relaxed.
2. I let my mind flow.

Rumination:

1. I ruminated (in the sense of revolving thoughts).
2. I tried to fight certain experiences.
3. I felt stressed.

**Semi-Structured Interview**

1. Does the participant ruminate sometimes?
2. If he/she ruminates
   1. Does he/she dwell on their thoughts? (Process: Dwelling of thoughts)
   2. Does the rumination persevere although the participant wants to stop it (Process: uncontrollability)
   3. Is the rumination directed towards the past? (Process: focus on past)
   4. What are the topics the participants usually ruminate on? (Thematic: open answer)
   5. How strong is the personal relevance from 0 to 100% (Thematic personal relevance)
   6. What emotions are related to the ruminations? Open answer
   7. Is the participant pessimistic during rumination (e.g. hopelessness)?
   8. How is the process of rumination described?
      1. Does a concrete action follow the ruminations?
      2. Does the rumination lead to solutions?
      3. Are there concrete thoughts during ruminations?
      4. Doe the participant think counterfactual during ruminations?
   9. How much time does the participant ruminate approximately per day?
   10. Is the participant affected by the ruminations (e.g. unable to do something else like reading a book)

**Means, standard deviations and correlations among assessed scales**

|  | Whole Sample | | HC | | MDD | |
| --- | --- | --- | --- | --- | --- | --- |
|  | mean | SD | mean | SD | mean | SD |
| VAS-Rum-pre | 2.31 | 2.53 | 0.67 | 0.75 | 3.96 | 2.62 |
| VAS-Rum-post | 3.15 | 2.59 | 1.56 | 1.31 | 4.73 | 2.60 |
| VAS-MW-Pre | 6.81 | 1.96 | 7.56 | 1.93 | 6.06 | 1.71 |
| VAS-MW-Post | 6.55 | 2.48 | 7.48 | 2.04 | 5.62 | 2.56 |
| ARSQ-Rum-Pre | 1.70 | 0.86 | 1.06 | 0.13 | 2.34 | 0.80 |
| ARSQ-Rum-Post | 1.87 | 0.93 | 1.16 | 0.34 | 2.58 | 0.78 |
| ARSQ-Self-Pre | 2.63 | 0.92 | 2.09 | 0.49 | 3.17 | 0.93 |
| ARSQ-Self-Post | 2.76 | 0.81 | 2.35 | 0.76 | 3.17 | 0.63 |
| ARSQ-Comf-Pre | 3.03 | 1.19 | 3.61 | 1.15 | 2.45 | 0.92 |
| ARSQ-Comf-Post | 2.57 | 0.95 | 3.08 | 0.83 | 2.07 | 0.80 |
| ARSQ-Plan-Pre | 2.39 | 0.78 | 2.41 | 0.68 | 2.37 | 0.89 |
| ARSQ-Plan-Post | 2.48 | 0.86 | 2.36 | 0.82 | 2.60 | 0.91 |
| ARSQ-Discon-Pre | 2.39 | 0.91 | 1.95 | 0.71 | 2.84 | 0.88 |
| ARSQ-Discon-Post | 2.50 | 0.92 | 1.93 | 0.62 | 3.07 | 0.83 |
| PANAS-NA-pre | 17.96 | 9.40 | 11.29 | 0.46 | 24.12 | 9.53 |
| PANAS-NA-post | 18.68 | 9.89 | 11.79 | 1.77 | 25.04 | 10.06 |
| PANAS-PA-pre | 26.10 | 7.83 | 30.58 | 6.61 | 21.96 | 6.56 |
| PANAS-PA-post | 24.00 | 8.33 | 28.13 | 8.62 | 20.19 | 6.03 |

Supplementary Table 1. Means and standard deviations of the used subscales and measures.

|  |  | 1 | 2 | 3 | 4 | 5 | 6 | 7 | 8 | 9 | 10 | 11 | 12 | 13 | 14 | 15 | 16 | 17 | 18 | 19 | 20 |
| --- | --- | --- | --- | --- | --- | --- | --- | --- | --- | --- | --- | --- | --- | --- | --- | --- | --- | --- | --- | --- | --- |
| 1 | BDI | 1 | .81^**^ | .79^**^ | .79^**^ | .60^**^ | .49^**^ | -.56 | -.53 | .10 | .16 | .55^**^ | .62^**^ | -.41^**^ | -.28^*^ | .69^**^ | .68^**^ | .74^**^ | .76^**^ | -.57^**^ | -.48^**^ |
| 2 | RRS | .81^**^ | 1 | .77^**^ | .77^**^ | .61^**^ | .49^**^ | -.43^**^ | -.53^**^ | .11 | .18 | .45^**^ | .53^**^ | -.49^**^ | -.22 | .78^**^ | .75^**^ | .59^**^ | .62^**^ | -.55^**^ | -.48^**^ |
| 3 | ARSQ-Rum-Pre | .79^**^ | .77^**^ | 1 | .86^**^ | .81^**^ | .50^**^ | -.50^**^ | -.48^**^ | .20 | .15 | .53^**^ | .58^**^ | -.50^**^ | -.18 | .83^**^ | .74^**^ | .77^**^ | .74^**^ | -.53^**^ | -.43^**^ |
| 4 | ARSQ-Rum-Post | .79^**^ | .77^**^ | .86^**^ | 1 | .70^**^ | .66^**^ | -.45^**^ | -.44*^*^ | .04 | .19 | .58^**^ | .67^**^ | -.43^**^ | -.17 | .78^**^ | .83^**^ | .74^**^ | .77^**^ | -.48^**^ | -.40^**^ |
| 5 | ARSQ-Self-Pre | .60^**^ | .61^**^ | .81^**^ | .70^**^ | 1 | .54^**^ | -.33^*^ | -.29^*^ | .29^*^ | .24 | .39^**^ | .46^**^ | -.38^**^ | -.14 | .79^**^ | .67^**^ | .61^**^ | .55^**^ | -.50^**^ | -.34^*^ |
| 6 | ARSQ-Self-Post | .49^**^ | .49^**^ | .50^**^ | .66^**^ | .54^**^ | 1 | -.25 | -.37^**^ | .13 | .34^*^ | .47^**^ | .60^**^ | -.28^*^ | -.19 | .57^**^ | .71^**^ | .57^**^ | .57^**^ | -.42^**^ | -.32^*^ |
| 7 | ARSQ-Comf-Pre | -.56^**^ | -.43^**^ | -.50^**^ | -.45^**^ | -.33^*^ | -.25 | 1 | .59^**^ | .14 | .05 | -.31^*^ | -.38^**^ | .46^**^ | .30^*^ | -.48^**^ | -.39^**^ | -.56^**^ | -.56^**^ | .53^**^ | .43^**^ |
| 8 | ARSQ-Comf-Post | -.53^**^ | -.53^**^ | -.48^**^ | -.44^**^ | -.29^*^ | -.37^**^ | .59^**^ | 1 | .01 | -.04 | -.42^**^ | -.37^**^ | .50^**^ | .45^**^ | -.46^**^ | -.51^**^ | -.56^**^ | -.60^**^ | .48^**^ | .58^**^ |
| 9 | ARSQ-Plan-Pre | .10 | .11 | .20 | .04 | .29^*^ | .13 | .14 | .01 | 1 | .66^**^ | .18 | .06 | .06 | .30^*^ | .20 | .23 | 0.02 | .00 | .02 | .07 |
| 10 | ARSQ-Plan-Post | .16 | .18 | .15 | .19 | .24 | .34^*^ | .05 | -.04 | .66^**^ | 1 | .10 | .21 | .05 | .09 | .23 | .35^*^ | 0.12 | .11 | .02 | .11 |
| 11 | ARSQ-Discon-Pre | .55^**^ | .45^**^ | .53^**^ | .58^**^ | .39^**^ | .47^**^ | -.31^*^ | -.42^**^ | .18 | .10 | 1 | .74^**^ | -.27 | -.06 | .49^**^ | .60^**^ | .48^**^ | .51^**^ | -.40^**^ | -.42^**^ |
| 12 | ARSQ-Discon-Post | .62^**^ | .53^**^ | .58^**^ | .67^**^ | .46^**^ | .60^**^ | -.38*^*^ | -.37^**^ | .06 | .21 | .74^**^ | 1 | -.31^*^ | -.08 | .57^**^ | .69^**^ | .58^**^ | .60^**^ | -.44^**^ | -.41^**^ |
| 13 | VAS-MW-Pre | -.41^**^ | -.49^**^ | -.50^**^ | -.43^**^ | -.38^**^ | -.28^*^ | .46^**^ | .50^**^ | .06 | .05 | -.27 | -.31^*^ | 1 | .41^**^ | -.45^**^ | -.36^**^ | -.52^**^ | -.49^**^ | .40^**^ | .28^*^ |
| 14 | VAS-MW-Post | -.28^*^ | -.22 | -.18 | -.17 | -.14 | -.19 | .30^*^ | .45^**^ | .30^*^ | .09 | -.06 | -.08 | .41^**^ | 1 | -.09 | -.14 | -.31^*^ | -.30^*^ | .39^**^ | .36^*^ |
| 15 | VAS-Rum-pre | .69^**^ | .78^**^ | .83^**^ | .78^**^ | .79^**^ | .57^**^ | -.48^**^ | -.46^**^ | .20 | .23 | .49^**^ | .57^**^ | -.45^**^ | -.09 | 1 | .86^**^ | .64^**^ | .60^**^ | -.60^**^ | -.50^**^ |
| 16 | VAS-Rum-post | .68^**^ | .75^**^ | .74^**^ | .83^**^ | .67^**^ | .71^**^ | -.39^**^ | -.51^**^ | .23 | .35^*^ | .60^**^ | .69^**^ | -.36^**^ | -.14 | .86^**^ | 1 | .68^**^ | .70^**^ | -.54^**^ | -.48^**^ |
| 17 | PANAS-NA-pre | .74^**^ | .59^**^ | .77^**^ | .74^**^ | .61^**^ | .57^**^ | -.56^**^ | -.56^**^ | .02 | .12 | .48^**^ | .58^**^ | -.52^**^ | -.31^*^ | .64^**^ | .68^**^ | 1 | .92^**^ | -.59^**^ | -.41^**^ |
| 18 | PANAS-NA-post | .76^**^ | .62^**^ | .74^**^ | .77^**^ | .55^**^ | .57^**^ | -.56^**^ | -.60^**^ | .00 | .11 | .51^**^ | .60^**^ | -.49^**^ | -.30^*^ | .60^**^ | .70^**^ | .92^**^ | 1 | -.51^**^ | -.45^**^ |
| 19 | PANAS-PA-pre | -.57^**^ | -.55^**^ | -.53^**^ | -.48^**^ | -.50^**^ | -.42^**^ | .53^**^ | .48^**^ | .02 | .02 | -.40^**^ | -.44^**^ | .40^**^ | .39^**^ | -.60^**^ | -.54^**^ | -.59^**^ | -.51^**^ | 1 | .82^**^ |
| 20 | PANAS-PA-post | -.48^**^ | -.48^**^ | -.43^**^ | -.40^**^ | -.34^*^ | -.32^*^ | .43^**^ | .58^**^ | .07 | .11 | -.42^**^ | -.41^**^ | .28^*^ | .36^*^ | -.50^**^ | -.48^**^ | -.41^**^ | -.45^**^ | .82^**^ | 1 |

Supplementary Table 2. Correlations between the used subscales and measures.
